# Usage of Fermental Traps for the Study of the Species Diversity of Coleoptera in Open Biotopes

**DOI:** 10.3390/insects14040404

**Published:** 2023-04-21

**Authors:** Alexander B. Ruchin, Leonid V. Egorov, Anatoliy A. Khapugin

**Affiliations:** 1 Joint Directorate of the Mordovia State Nature Reserve and National Park “Smolny”, 430005 Saransk, Russia; 2 Prisursky State Nature Reserve, 428034 Cheboksary, Russia; 3 Institute of Environmental and Agricultural Biology (X-BIO), Tyumen State University, 625003 Tyumen, Russia

**Keywords:** abundance, fermental traps, beer traps, Coleoptera, fauna, biodiversity, occurrence

## Abstract

**Simple Summary:**

The possibilities of using beer traps for the study of Coleoptera fauna in various open biotopes were studied. The biodiversity of beetles was 208 species from 35 families. The largest number of species belonged to the families Cerambycidae (35 species), Curculionidae (26 species), and Elateridae (25 species). Only 13 species were found to be common to all habitats. Some patterns of species distribution in biotopes were revealed. The maximum species diversity with the greatest equalization of species was characteristic of meadows. We recommend the use of beer traps for ecological studies of Coleoptera fauna in open biotopes.

**Abstract:**

Usage of a variety of non-trivial ways to study Coleoptera gives unexpected and original results. The studies were conducted using simple traps with fermenting baits in the central part of European Russia. There were 286 trap exposures, and 7906 Coleoptera specimens (208 species from 35 families) were collected. The largest number of species belonged to the families Cerambycidae (35 species), Curculionidae (26 species), and Elateridae (25 species). One species each was noted in 12 families. Traps were applied in five open habitats (dry meadow, shore, floodplain meadow, cuttings under power lines, and glade in woods). Only 13 species were common to all habitats: *Cetonia aurata*, *Protaetia marmorata*, *Dasytes niger*, *Cryptarcha strigata*, *Glischrochilus grandis*, *Glischrochilus hortensis*, *Glischrochilus quadrisignatus*, *Soronia grisea*, *Notoxus monoceros*, *Aromia moschata*, *Leptura quadrifasciata*, *Rhagium mordax*, *Anisandrus dispar*. Dry meadows were dominated by *C*. *aurata*, *A*. *murinus*, and *P*. *cuprea volhyniensis*. A shore was dominated by *C*. *strigata*, *G*. *grandis*, *G*. *hortensis*, *S*. *grisea*, and *A*. *dispar*. The dominant species in floodplain meadows were *G*. *hortensis*, *S*. *grisea*, and *A*. *dispar*. On cuttings under power lines, the most numerous were *C*. *aurata*, *P*. *cuprea volhyniensis*, and *C*. *viridissima*. In forest glades, the maximum abundance data were obtained for *G*. *grandis*, *C*. *strigata*, and *A*. *dispar*. The Shannon index was greatest in meadow habitats of varying degrees of moisture, while it was minimal on the shore. The increase in the Simpson index was also characteristic of the shore. These data indicate reduced species diversity combined with the dominance of several species in this biotope. The maximum species diversity with the highest level of species alignment was characteristic of meadow plots, while lower values were obtained under power lines and in forest glades. We recommend the usage of fermental traps with beer for ecological studies of the Coleoptera fauna in open biotopes.

## 1. Introduction

A variety of methods are used to collect information on insect biodiversity in ecosystems. Entomological net mowing, light traps, window traps, barrier traps, pitfall traps, and Malaise traps are commonly available and routinely used methods [1,2,3,4,5,6,7,8]. Each of these methods has both advantages and disadvantages. For example, manual collection of insects with a net is limited by the availability of individual groups at the observer level with the net extended, the activity of the groups, the secretive lifestyle of some insects, and other aspects. Light traps require electricity, as well as certain groups of special lamps with a dedicated spectrum. In addition, not every method of catching can be used for certain research purposes, for example, to study seasonal phenomena, migrations, and vertical stratifications, as well as the inability to use traps in some biotopes [5,9,10,11].

Traps of various types use a variety of substrates (e.g., adult and larval food items) and chemicals (pheromones, attractants) as baiting agents to catch insects. By applying unusual collection methods with different baits, it is possible to detect new species that have not previously been recorded using conventional insect survey methods. The use of decaying fruit or vegetable baits to study insect communities has been practiced in many countries [10,12,13,14]. Baits in the form of fermenting liquids, such as wine and beer, with the addition of bananas, apples, sugar, and other substrates, have shown to be effective for the detection of many insects [15,16,17,18,19,20].

One of the most widespread and numerous groups of insects in the world is the order Coleoptera [21]. They include a variety of ecological groups, such as predators, phytophages, saprophages, and others [22,23,24]. The significant diversity of Coleoptera allowed them to occupy a wide variety of biotopes in all ecosystems of the globe. These include species that prefer forest ecosystems, as well as species more commonly found in open habitats [25,26,27,28,29]. The significant contribution that Coleoptera make to the functioning and biodiversity of open ecosystems is particularly noteworthy. Such systems include meadows of various types, farmlands, glades, cuttings, clearings, and other parts of the landscape. Common to these ecosystems is the absence or small amount of trees and shrubs, and a well-developed herbaceous tier. In such ecosystems, the choice of study methods is not very significant, because the main vigorous activity of Coleoptera occurs in the soil and herbaceous tiers. On the other hand, many flying Coleoptera species are capable of active movements and cannot always be accounted for during studies. In such cases, full-fledged studies can be carried out with the usage of traps with baits; there are examples of such experiments. Some Cerambycidae were caught in open biotopes at different distances from the forest, and traps with attractants collected more individuals [30]. In cuttings, the Coleoptera species diversity increased in the third year after logging [31]. A combination of open and closed biotopes has been shown to play a positive role in increasing the abundance and species diversity of dung beetles using bait traps [32].

The present study is aimed at developing and applying an approach consisting of the use of traps with bait beer and sugar (beer traps) as the baits in open habitats. The following research tasks were established: (i) to develop the methods of the use of beer traps in open habitats; (ii) to determine the possible use of beer traps for revealing the Coleoptera diversity in open habitats; (iii) to investigate the diversity of Coleoptera communities in various habitats using beer traps.

## 2. Materials and Methods

### 2.1. Placement of Traps

Each trap was a plastic 5 L container with a window cut out of it on one side at a distance of 10 cm from the bottom. Beer with an addition of honey, jam, or sugar was used as bait [33].

**Figure 1:**
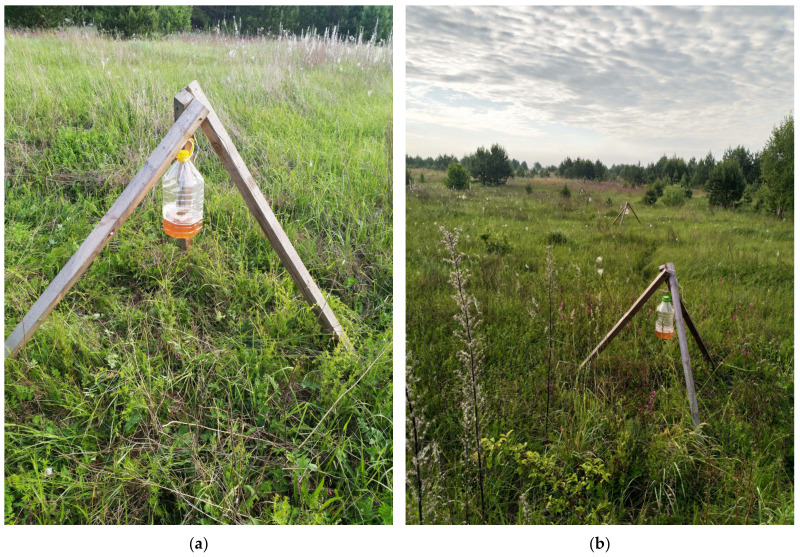
Different options for installing beer traps in open biotopes. (**a**) Single trap; (**b**) Installation of four traps in a row.

The traps were placed on wooden tripods at a height of 1.5 m above the soil surface, which corresponds to the optimal height of beetle flight [34]. Thus, in open ecosystems, the trap was located above the level of the herbaceous tier. The tripods were placed singly or in groups of 3–4 in a row. The distance between the tripods in groups was 10–12 m (Figure 1).

The following types of open biotopes were chosen for study: dry meadow, floodplain meadow, shore, cuttings under power lines, forest glade. Each habitat differed from the others in a number of features, which are described in Table 1.

Several terms were used to determine the effectiveness of the traps. (1) Occurrence—the ratio of the number of samples where a species (taxonomic group) is present to the total number of samples (expressed in %). In the analysis, we used data on the number of all Coleoptera individuals in the traps for the exposure time. (2) Exposure time—the period between hanging a trap and taking samples for analysis (expressed in days). The exposure of the traps ranged from 7 to 15 days. The contents were removed from the traps and placed in a jar of alcohol. The laboratory analyzed the contents and identified the samples.

### 2.2. Identification

The lists of species within families are given using contemporary data [35,36]. The nomenclature is specified according to the *Catalogue of Palaearctic Coleoptera* [37,38,39,40,41,42,43,44,45], as well as according to other publications [46,47]. The years of description of some beetle species are given by Bousquet [48].

### 2.3. Data Analyses

Saproxylic species were determined with guidance from publications [49,50,51,52] and our own data. Anthophilic species were considered to be species that repeatedly visited flowers. In this case, we used our own long-term observations, as well as information from publications [50,51,53].

We used the Jaccard index to compare the Coleoptera fauna between habitats. The Jaccard similarity index was calculated for all study plots. We also calculated the Shannon index and the Simpson index [54,55] to understand the species diversity and community alignment. Yet, in our calculations, we did not take into account insects that were not identified at the species level.

The ordination techniques, using principal component analysis (PCA), defined the major gradients in the arrangement of the studied species selected for the analysis of the studied habitats. For the ecological interpretation of the ordination axes, groups of the studied habitats (based on the species abundance) were plotted in the PCA ordination diagram as supplementary environmental data. We analyzed the species, which were represented by at least 100 exemplars during the sampling period. In addition, we used the coefficient of determination (R^2^, or R-squared). Individual-based rarefaction was performed to compare the species diversity of the Coleoptera species in open habitats. It was used to investigate the richness of the Coleoptera community expected in a study plot based on collected individuals. Hill numbers included the three most widely used species diversity measures, namely, the species richness (S), the exponential of Shannon entropy (exp(H)), and the inverse of the Simpson concentration index (1/D) [55]. All statistical analyses were carried out using PAST 4.07 [56].

## 3. Results

**Figure 2:**
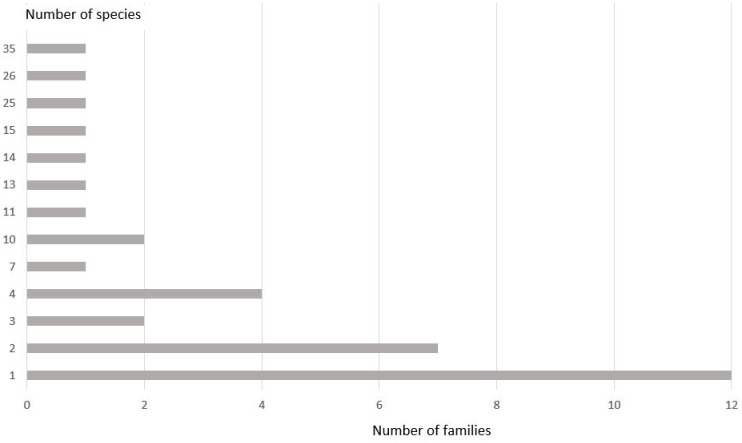
Distribution of Coleoptera families by the number of captured species in the beer traps.

During the study, 286 trap exposures were made, and 7906 Coleoptera specimens were collected (Appendix A). In total, 208 species from 35 families were recorded in all biotopes (Figure 2, Table 2). The largest number of species found in the traps belonged to the families Cerambycidae (35 species), Curculionidae (26 species), and Elateridae (25 species). Only one species was recorded among 12 families (Throscidae, Lycidae, Hydrochidae, Anthicidae, Melandryidae, Mycetophagidae, Latridiidae, Monotomidae, Cucujidae, Phalacridae, Laemophloeidae, and Attelabidae); two species were recorded among seven families (Buprestidae, Staphylinidae, Cleridae, Mordellidae, Scraptiidae, Erotylidae, and Anthribidae). The highest total number of collected specimens were found for the following families: Nitidulidae (2969 specimens, 37.5%), Scarabaeidae (1680, 21.2%), Cerambycidae (801, 10.1%), and Curculionidae (719, 9.1%) (Appendix A).

**Figure 3:**
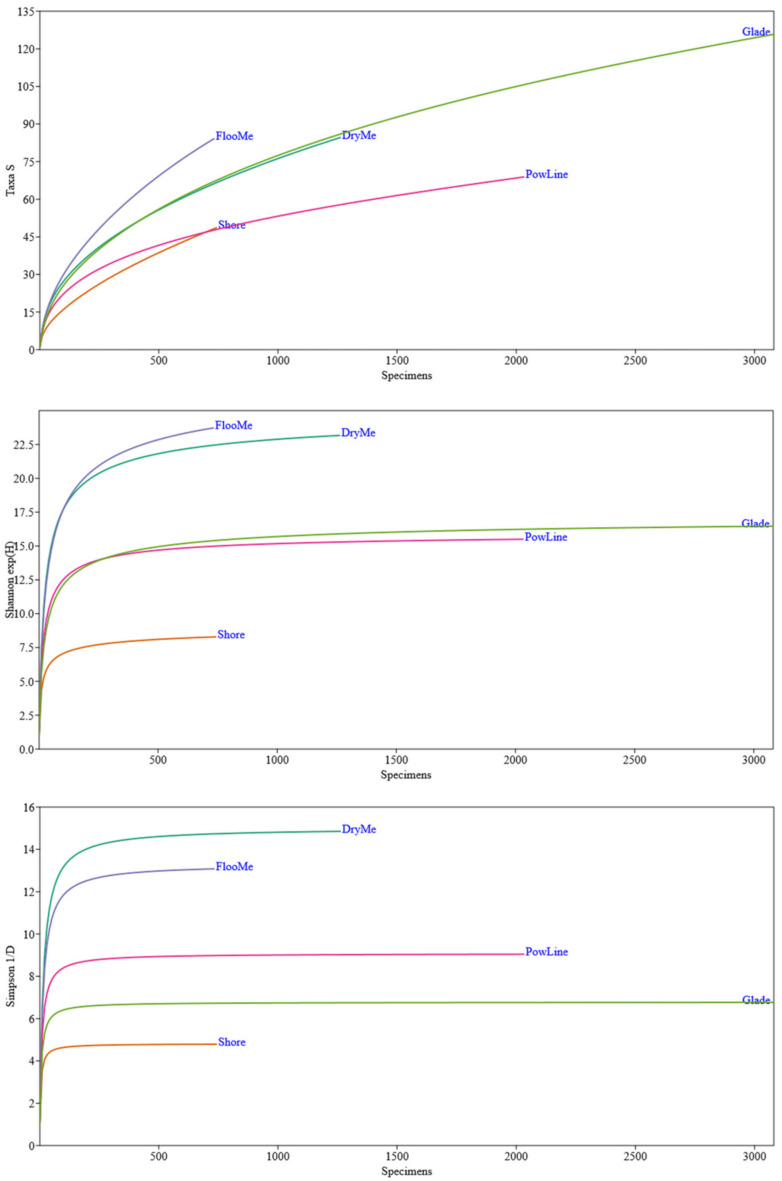
Differences in species richness (Taxs S) (**top**), exponential of Shannon entropy (exp(H)) (**middle**), and inverse Simpson index (1/D) (**bottom**) of beetles in five habitats. Designations: DryMe—dry meadows, Shore—shores, FlooMe—floodplain meadows, PowLine—cuttings under power lines, Glade—forest glade.

All parameters of the α-diversity of Coleoptera (i.e., the species richness, exponential of Shannon entropy, and inverse of the Simpson index) were significantly different between various habitat types. The species richness and the exponential of Shannon entropy were significantly higher in floodplain meadows. At the same time, the inverse of the Simpson index was considerably higher in dry meadows (Figure 3). Our data show that the usage of the beer traps to compare the Coleoptera biodiversity in open biotopes gives interesting results. The species diversity and abundance of Coleoptera differ by biotope types (Table 2). The highest total abundance of Coleoptera was characteristic of glades. The maximum species diversity was also noted there. It might seem that this is due to the significant number of traps that were installed in such habitats. However, 28% more traps were installed in DryMe. Yet, the species diversity in that habitat was 46% lower, and the total abundance of individuals was 2.4 times lower. A similar number of specimens were caught in FlooMe and Shore. However, the species diversity between these habitats differed almost twice (Table 2). The Shannon index was the greatest in meadow habitats of varying degrees of moisture, while it was minimal on the shore. The increase in the Simpson index was also characteristic of Shore. The maximum species diversity with the highest level of species alignment was characteristic of meadow plots, while lower values were obtained under power lines and in forest glades.

In a study of 5 different habitats, out of 208 species, only 13 species (4.5%) were found to be common to them: *Cetonia aurata* (Linnaeus, 1758), *Protaetia marmorata* (Fabricus, 1792), *Dasytes niger* (Linnaeus, 1761), *Cryptarcha strigata* (Fabricius, 1787), *Glischrochilus grandis* (Tournier, 1872), *Glischrochilus hortensis* (Geoffroy, 1785), *Glischrochilus quadrisignatus* (Say, 1835), *Soronia grisea* (Linnaeus, 1758), *Notoxus monoceros* (Linnaeus, 1761), *Aromia moschata* (Linnaeus, 1758), *Leptura quadrifasciata* Linnaeus, 1758, *Rhagium mordax* (De Geer, 1775), *Anisandrus dispar* (Fabricius, 1792). The remaining species (273 species, 95.5%) were not found in all biotopes. Of the total diversity, a significant number of species (113, 39.5%) were found only in one habitat type and were no longer found in other habitats.

**Figure 4:**
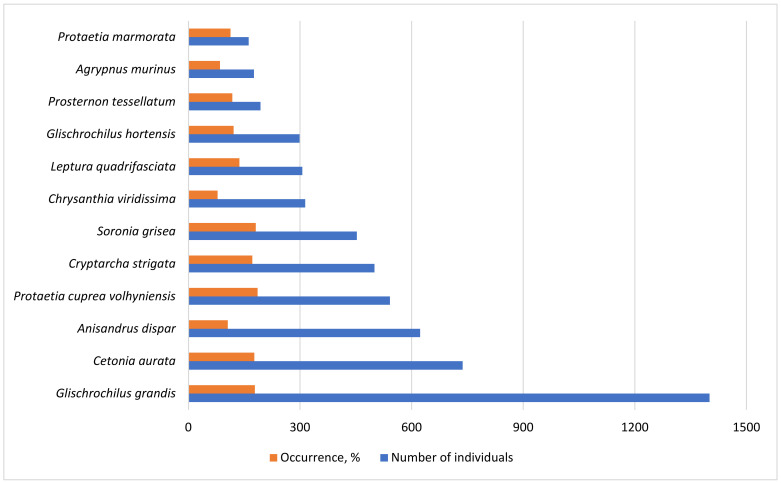
Total occurrence and abundance of Coleoptera species present in all biotopes in the beer traps during the entire observation period.

In terms of the total number and common occurrence, 12 species predominated: *C*. *aurata*, *Agrypnus murinus* (Linnaeus, 1758), *P*. *marmorata*, *Protaetia cuprea volhyniensis* (Gory & Percheron, 1833), *Prosternon tesselatum* (Linnaeus, 1758), *C*. *strigata*, *G*. *grandis*, *G*. *hortensis*, *S*. *grisea*, *Chrysanthia viridissima* (Linnaeus, 1758), *L*. *quadrifasciata*, *A*. *dispar* (Figure 4). They accounted for 72.2% of the total number of Coleoptera in all traps. The highest total number of catches was *G*. *grandis*; however, the occurrence of this species in the traps was lower than that of *P*. *cuprea volhyniensis* and *S*. *grisea*. As a result, these three species had the highest total occurrence in catches in all biotopes.

**Figure 5:**
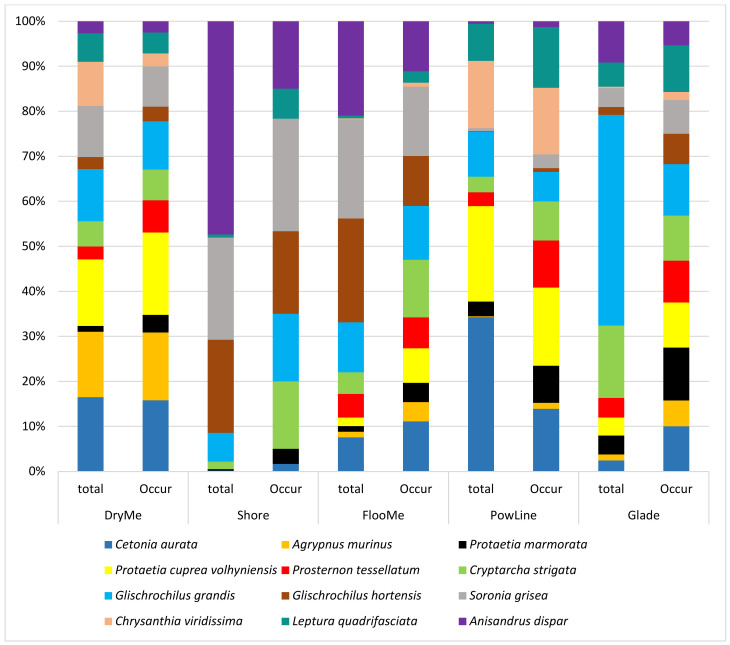
Absolute abundance and total occurrence of some Coleoptera species in selected biotopes.

We analyzed the abundance and occurrence of these 12 species in different habitats (Figure 5). In dry meadows, *C*. *aurata*, *A*. *murinus*, and *P*. *cuprea volhyniensis* dominated among these species, and these same species occurred more frequently than others. Species of the family Nitidulidae (*C*. *strigata*, *G*. *grandis*, *G*. *hortensis*, *S*. *grisea*), as well as *A*. *dispar*, dominated in number on the shore. The dominant species in floodplain meadows were *G*. *hortensis*, *S*. *grisea*, and *A*. *dispar*. In cuttings under power lines, the most numerous were *C*. *aurata*, *P*. *cuprea volhyniensis*, and *C*. *viridissima*. In forest glades, the maximum abundance data were obtained for *G*. *grandis*, *C*. *strigata*, and *A*. *dispar*.

The results showed that under power lines and in glades, the relative number of saproxylic species was higher than in other habitats. At the same time, there were more anthophilic Coleoptera species under power lines and in meadows (Table 2).

**Figure 6:**
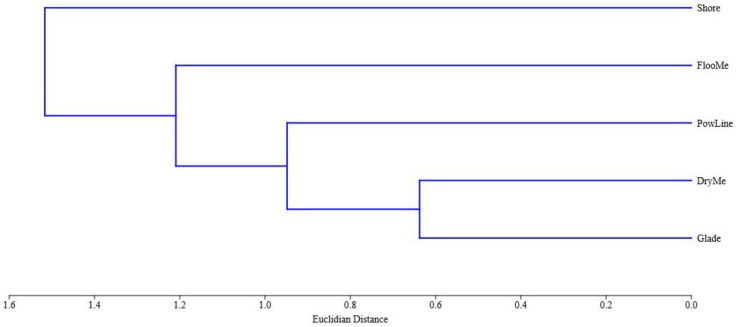
Similarity of Coleoptera species composition between different open habitats based on the Jaccard index.

The Jaccard index revealed several clusters based on Coleoptera species similarity (Figure 6). The greatest similarity was obtained when comparing glade in woods and dry meadow. The habitat most different from the other habitats turned out to be the shore.

**Figure 7:**
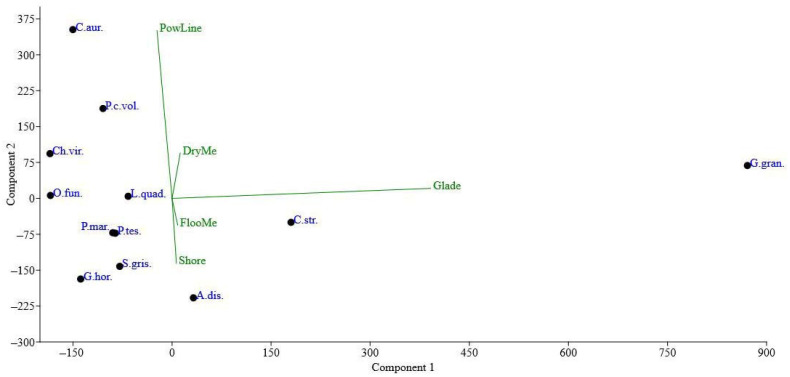
Principal component analysis (PCA) ordination diagram of the selected Coleoptera species based on their abundance in the studied habitats. Species: C.aur.—*Cetonia aurata* (Scarabaeidae), A.mur.—*Agrypnus murinus* (Elateridae), P.mar.—*Protaetia marmorata* (Scarabaeidae), P.c.vol.—*Protaetia cuprea volhyniensis* (Scarabaeidae), P.tes.—*Prosternon tessellatum* (Elateridae), C.str.—*Cryptarcha strigata* (Nitidulidae), G.gran.—*Glischrochilus grandis* (Nitidulidae), G.hor.—*Glischrochilus hortensis* (Nitidulidae), S.gris.—*Soronia grisea* (Nitidulidae), Ch.vir.—*Chrysanthia viridissima* (Oedemeridae), L.quad.—*Leptura quadrifasciata* (Cerambycidae), A.dis.—*Anisandrus dispar* (Curculionidae).

Figure 7 shows that among the selected Coleoptera species, *G*. *grandis* differs significantly due to the highest abundance of this species in all habitats. In forest glades, its abundance was the most considerable. Other species have fewer differences among the studied habitats. Thus, *C*. *aurata* and *P*. *cuprea volhyniensis* were more abundant along power lines. In contrast, *C*. *strigata* and *A*. *dispar* were less abundant in habitats of power lines. Of them, *C*. *strigata* had a relatively high abundance in forest glades, while *A*. *dispar* on river banks. Other species show no remarkable differences among habitats, as they were less represented in forest glades.

## 4. Discussion

Fermenting baits, consisting of aqueous mixtures of sugar and beer that are allowed to ferment, release volatile substances that attract certain species of wood insects. The experience of using the fermenting (beer) traps by different researchers has shown that different groups of insects are attracted to such baits, which have been used in forests and hung from tree branches most often and for a long time [15,57,58,59,60]. These baits are another source of attractants for some tree insects. They were thought to attract those insects that normally feed on tree secretions (or on tree trunks and branches) or honeydew [3,61,62,63,64]. However, as the practice of application and the analysis of species diversity has shown, species that are flower-visiting, i.e., feed on nectar, pollen, etc., are found in the traps [33,64,65,66]. Thus, in previous studies, the fermenting (beer) traps were used in forest ecosystems, sparse forests, or in those biotopes with shrubs and undergrowth trees.

Our study attempted to use the beer traps to catch Coleoptera in open biotopes, which are spaces without trees or with small bushes. Simple tripods, on which traps were placed, were used for this purpose. Such traps were located above the grass cover of ecosystems, which ensured their successful use. The usage of the beer traps in open habitats was found to provide good information on Coleoptera biodiversity, species abundance and occurrence, species biology, and seasonal dynamics. During the experiments, 208 species from 35 families were collected, which indicates the advisability of using this method to study Coleoptera communities.

When comparing the results, it turned out that the total number and the greatest species diversity of Coleoptera were characteristic of glades in the forest. In second place for these indicators was PowLine. The surrounding forested areas had some influence on the Coleoptera species diversity in these habitats. Species that are characteristic of forest ecosystems were recorded in the traps. For example, the forest species *Cryptarcha strigata* and *Glischrochilus grandis* dominate significantly in the forest glades. The meadows are dominated by *Cetonia aurata* and *Protaetia cuprea volhyniensis*, which also dominate PowLine; these species are more characteristic of open ecosystems. Apparently, because of this, they were in different clusters, according to the Jaccard index.

Interestingly, the highest biodiversity of Coleoptera, coupled with the highest species alignment, was characteristic of meadows. On the shore, the Shannon index is minimal, and the Simpson index is maximal. These data indicate reduced species diversity, combined with the dominance of several species in this biotope (two species of Nitidulidae, as well as *Anisandrus dispar*, dominated this biotope).

Of the total number of species that were attracted to the fermental traps, let us single out those that were particularly frequent in the traps, yet abundant in various open habitats.

The most common species was *Cetonia aurata* (Scarabaeidae; the average occurrence for all biotopes is 35.4%). This species lives in a wide range of biotopes. The species is anthophilic and occurs on flowers of plants from the families Umbelliferae, Rosacea, and Asteraceae [67,68]. Larvae develop in decaying wood and decaying organic substrates [69]. Previously, it was shown that the species prefers fermental traps located at low altitudes [65,70].

*Oxythyrea funesta* (Scarabaeidae; the average occurrence for all biotopes is 16.8%) occurs in various open habitats rich in herbaceous vegetation (in glades, meadows, cuttings, and roadsides). It is not uncommon in orchards, where it can damage the reproductive parts of trees and shrubs [70,71,72], and it is rarely found inside forested areas under tree crowns and in undergrowth. In our studies, the highest occurrence and significant abundance were noted in cuttings under power lines.

*Protaetia marmorata* (Scarabaeidae; the average occurrence for all biotopes is 22.6%) inhabits a variety of forest types and is found in parks, orchards, forest shelter belts, and other habitats [49]. It is usually one of the most numerous species of the genus. It occurred more often in the traps at 7–12 m, and to a lesser extent in the lower forest tiers [68,73,74], which suggests that it is confined to the upper forest tiers. According to our observations, it often occurs on tree trunks, where it feeds on sap flowing out. Larval development was observed in the hollows of dead deciduous trees [75,76].

*Protaetia cuprea volhyniensis* (Scarabaeidae; the average occurrence for all biotopes is 37.2%) inhabits a wide variety of ecosystems. It prefers different types of forests [66,77,78]; however, judging by our results, it is also not uncommon in open biotopes. In comparison, the occurrence of this species in forest ecosystems was two times lower than in open ecosystems [65]. Larvae usually develop in active and abandoned anthills, sometimes in sawdust and piles of garbage [79,80]. It is quite often found on flowering plants. Perhaps its frequent occurrence in open biotopes can be explained by its anthophilic nature.

*Agrypnus murinus* (Elateridae; the average occurrence for all biotopes is 17.0%) occurs in fields, meadows, orchards, and other open habitats [81,82]. Adults are herbivores, but are regularly observed on flowers in spring and summer. Larvae feed on roots, but can also be carnivorous [83,84]. Earlier in the beer traps located in forest ecosystems, it was registered very rarely [65,68,74]. In our studies, it was not found only on a sandy spit. In dry meadows, the species occurred very often in high abundance.

*Prosternon tessellatum* (Elateridae; the average occurrence for all biotopes is 23.6%) is one of the most common species of the Elateridae family in the center of European Russia. This is a eurytopic species that lives in a wide variety of biotopes [81,85]. On the edges, glades, and meadows, this species is very common, and it is often seen on flowering plants. Previously, it was recorded very rarely in the fermental traps located in forest ecosystems [65,68,74]. In our studies, the maximum abundance and frequent occurrence were typical for glades and cuttings under power lines.

*Trichodes apiarius* (Cleridae; the average occurrence for all biotopes is 15.7%) inhabits glades, edges, and meadows. Adults are found on flowers, while larvae are associated with Hymenoptera nests, especially solitary bees. Living on flowers, the species consumes pollen [86,87]. In our studies, the highest occurrence and significant abundance were noted in cuttings under power lines.

*Cryptarcha strigata* (Nitidulidae; the average occurrence for all biotopes is 34.4%) is often found in deciduous and mixed forests. Adults feed on the flowing sap of oaks. Larvae develop on the bark in these places [88]. It occurred frequently in the beer traps, sometimes with very significant numbers. Earlier, it was shown that the occurrence of this species in the beer traps exceeded 50% [65]. In this study, we obtained significantly lower values. We believe that *C*. *strigata* is a forest species that prefers closed habitats.

*Glischrochilus grandis* (Nitidulidae; the average occurrence for all biotopes is 35.8%) has a biology that is partly similar to the previous species. However, it is known that *G*. *grandis* also develops in fungi and rotten berries, as well as on various decaying substrates [65,89]. It is the most abundant species in our studies. Especially large numbers were observed in forest glades.

*Glischrochilus hortensis* (Nitidulidae; the average occurrence for all biotopes is 24.3%) occurs more often in deciduous and mixed forests. Adults can often be found on the flowing sap of oaks and under the bark of fallen and dying deciduous trees, where larvae develop. Larvae can also develop in rotten fruits and vegetables [65,88,90].

*Soronia grisea* (Nitidulidae; the average occurrence for all biotopes is 36.3%) lives in forest ecosystems, where it can be found both on the edges and in the interior of the forest [65,68,88]. It is often caught on beer baits in various forests [65,68,73,91]. In terms of seasonal dynamics, there is an increase in the number in May–June, but single specimens are found throughout the season [92]. According to new data, this species is also well attracted to beer traps in open habitats. Its abundance and occurrence in all studied biotopes were high.

*Chrysanthia viridissima* (Oedemeridae; the average occurrence for all biotopes is 15.7%) occurs in various meadows, roadsides, cuttings, and edges, and sometimes in agrocenoses and other open habitats. In forest ecosystems, it prefers open areas: glades, cuttings, forest roads [65,74]. The species is often found on various plants, where it feeds on pollen [93]. In our studies, the greatest occurrence was characteristic of cuttings under power lines.

*Leptura quadrifasciata* (Cerambycidae; the average occurrence for all biotopes is 27.4%) occurs in a variety of habitats, but prefers open areas with well-developed flowering herbaceous vegetation. It is often caught in fermental traps inside forested areas [68,73,74]. The larvae of this species develop in dead or decaying hardwood [94]. Adults are anthophilic. In our studies, it was most often found in forest glades and on cuttings under power lines.

*Notoxus monoceros* (Anthicidae; the average occurrence for all biotopes is 5.9%) was found in all biotopes. It usually lives in open habitats with sparse herbaceous vegetation. This species is often found on flowering plants [95]. Interestingly, the species is attracted to a variety of baits based on vinegar and alcohol [96,97]. The occurrence of the species was low, but the beetles were most often found in forest glades.

*Anisandrus dispar* (Curculionidae; the average occurrence for all biotopes is 21.1%) is found in various forest habitats. It is a pest of forest plantations (chestnut, oak, beech, elm, poplar, etc.) and also inhabits many fruit plants (apple, pear, apricot, plum, peach, walnut, hazelnut). It enters into a complex symbiosis with a fungus (*Ambrosiella hartigii*), which allows the larvae to develop in wood tissues that are poor in nutrients [98]. Mass departure falls in the spring months [92,99]. It is often attracted to the fermental traps. Apparently, it is baited by ethanol, which is known to be used in traps to control this pest [100,101].

The results showed that the number of saproxylic and anthophilic Coleoptera species collected by the fermental traps in open biotopes was quite high. However, the number of saproxylic species was less than noted in other studies in forest ecosystems [65,68,73]; yet, at the same time, the number of anthophilic species, on the contrary, increased. The highest numbers of anthophilic species were in the families of Scarabaeidae, Cerambycidae, and Curculionidae. The same families lead in terms of the total number of species. The family Nitidulidae is noteworthy, since it includes anthophilic species (e.g., *Cychramus luteus* and *Glischrochilus grandis*); however, beer traps allowed us to collect the species, which are not anthophilic ones. The main feeding source of their nutrition on the imago stage are sweet secretions (juice) on the trunks of *Quercus robur*, *Populus tremula*, and *Acer platanoides*. The number of such species (e.g., *Glischrochilus hortensis*, *Cryptarcha strigata*, and *Soronia grisea*) in beer traps was considerably higher than other species of this family. This indicates that this type of bait can be quite successfully used in open habitats for species that are pollenophagous, nectarophagous, and flower-visiting.

## 5. Conclusions

The traps used in this study can be made in a short time using readily available materials. They are easy to maintain and install, and the bait is easy to make. The diversity of Coleoptera that are attracted to such traps is considerable (208 species from 35 families). Most families are represented by 1–3 species. The largest number of species found in the traps belong to the families Cerambycidae, Curculionidae, and Elateridae. The families of Nitidulidae, Scarabaeidae, Cerambycidae, and Curculionidae had the highest total number of collected specimens. Beetles of these families were mainly anthophilic and/or juice-feeding species. Five open habitats were studied using the fermental traps at low altitude. Differences in the abundance and occurrence of species in habitats, the species diversity of communities, and species dominance were revealed. Thus, the usage of fermenting bait based on beer and sugar in open biotopes is expedient and reflects the Coleoptera fauna well in these communities, as it does in forest ecosystems. Similar studies in open biotopes should be carried out during the entire season of insect activity. We recommend the usage of the fermental traps with beer for ecological studies of the Coleoptera fauna in open biotopes. This method can be used to study the seasonal dynamics of species, the habitat preferences of individual species, the abundance and dynamics of communities, and the biotopic features of fauna.

## Figures and Tables

**Table 1:** Summary of the characteristics of the studied habitats.

No.	Biotopes	Brief Description
1	Dry meadow (DryMe)	It is a meadow with well-developed vegetation. The percent cover is about 80%. There are open ground gaps without herb cover. The vegetation is represented predominantly by grasses, with some ruderal plants (e.g., *Agrimonia eupatoria*, *Cichorium intybus*, and *Erigeron annuus*). The height of the herb cover is about 30 cm. The study site is surrounded by pine (*Pinus sylvestris*) undergrowth of 1.0–3.0 m height.
2	Shore (Shore)	It is a river sand bank, formed due to the river sediments of the sand. This site is 0.8 ha. Beer traps are located 5 m from the water. On one side, this site borders the river; on other sides, the site is surrounded by willow thickets. The soil is sandy and highly mobile. The herb vegetation is slightly pronounced, represented mainly by communities of *Petasites spurius*.
3	Floodplain meadow (FlooMe)	It is a floodplain meadow site with well-developed vegetation and sufficient humidity. The percent cover is about 95–100%. There are almost no open gaps of the ground. The height of the vegetation is up to 50–60 cm. The vegetation is represented mainly by cereal and forbs species.
4	Cuttings under power lines (PowLine)	In these habitats, cutting of trees and shrubs is constantly carried out to protect power lines. These habitats stretched along power lines are surrounded by forest ecosystems, mainly consisting of *Pinus sylvestris* and *Betula pendula*, with the participation of other deciduous species of trees and shrubs. In contrast to forest glades, these habitats have a linear allocation of open space (along power lines). The width of the open strip is 180–200 m. Low shrubs occur in some sites. Herb vegetation is very well expressed. The percent cover is up to 80%. There are open areas with bare soil here.
5	Forest glade (Glade)	These are open habitats surrounded by mixed or deciduous forests on all sides. The area ranges from 0.02 ha to 0.95 ha. Herb vegetation is well expressed. The percent cover is up to 90–100%. Areas with bare soil are few.

**Table 2:** Summary data from studies in 2020–2022.

	DryMe	Shore	FlooMe	PowLine	Glade	Total
Number of traps	90	20	50	56	70	286
Total of individuals	1275	751	747	2040	3093	7906
Shannon index	3.06	1.88	3.06	2.69	2.62	
Simpson index	0.07	0.25	0.09	0.11	0.18	
Number of species (excluding unidentified ones)	81	43	80	66	118	208
Number of saproxylic	44	23	34	45	67	106
Number of saproxylic species (% of the total number of species per biotope)	54.3	53.5	42.5	68.2	56.8	51.0
Number of anthophilic	44	19	42	45	43	91
Number of anthophilic species (% of the total number of species per biotope)	54.3	44.2	52.5	68.2	36.4	43.8
Total of families	21	18	23	19	31	35

## Data Availability

Not applicable.

## Appendix A

**Table A1:** Biodiversity and occurrence of Coleoptera from fermental traps in the European part of Russia (N—Number of specimens, O—occurrence, %).

Family, Species	DryMe		Shore		FlooMe		PowLine		Glade		Total
	N	O	N	O	N	O	N	O	N	O	
**Carabidae**											
*Agonum lugens* (Duftschmid, 1812)					1	2.0			1	1.4	2
*Amara aenea* (De Geer, 1774)	1	1.1									1
*Amara tibialis* (Paykull, 1798)	2	2.2			1	2.0					3
*Cicindela hybrida* (Linnaeus, 1758)			2	5.0							2
*Calathus fuscipes* (Goeze, 1777)					1	2.0					1
*Calathus melanocephalus* (Linnaeus, 1758)	4	2.2			1	2.0			1	1.4	6
*Harpalus luteicornis* (Duftschmid, 1812)	1	1.1									1
*Harpalus signaticornis* (Duftschmid, 1812)	1	1.1									1
*Lebia cruxminor* (Linnaeus, 1758)	1	1.1					1	1.8			2
*Lebia marginata* (Geoffroy, 1785)									1	1.4	1
**Buprestidae**											
*Agrilus* sp.			1	5.0							1
*Anthaxia quadripunctata* (Linnaeus, 1758)					2	4.0	4	5.4	3	4.3	9
*Chrysobothris chrysostigma* (Linnaeus, 1758)					2	4.0	1	1.8			3
**Throscidae**											
*Trixagus* sp.									3	4.3	3
**Lycidae**											
*Lygistopterus sanguineus* (Linnaeus, 1758)	1	1.1									1
**Cantharidae**											
*Cantharis fusca* (Linnaeus, 1758)	1	1.1							10	5.7	11
*Cantharis livida* (Linnaeus, 1758)			1	5.0							1
*Cantharis obscura* (Linnaeus, 1758)									2	1.4	2
*Cantharis nigricans* (O.F. Müller, 1776)	2	2.2			1	2.0			2	2.8	5
*Cantharis figurata* (Mannerheim, 1843)			1	5.0	3	4.0					4
*Cantharis pellucida* (Fabricius, 1792)	1	1.1									1
*Cantharis rustica* (Fallén, 1807)	3	2.2							7	5.7	10
*Rhagonycha lignosa* (O.F. Müller, 1764)					1	2.0					1
*Rhagonycha nigripes* (W. Redtenbacher, 1842)							1	1.8	1	1.4	2
*Rhagonycha nigriventris* (Motschulsky, 1860)					3	6.0					3
**Elateridae**											
*Actenicerus sjaelandicus* (O.F. Müller, 1764)	1	1.1			1	2.0					2
*Agriotes lineatus* (Linnaeus, 1767)	1	1.1							1	1.4	2
*Agriotes sputator* (Linnaeus, 1758)					1	2.0					1
*Agrypnus murinus* (Linnaeus, 1758)	137	46.7			6	10.0	3	5.4	30	22.9	176
*Ampedus balteatus* (Linnaeus, 1758)									1	1.4	1
*Ampedus cinnabarinus* (Eschscholtz, 1829)	3	3.3					1	1.8	6	4.3	10
*Ampedus elongatulus* (Fabricius, 1787)	7	3.3									7
*Ampedus nigroflavus* (Goeze, 1777)	1	1.1									1
*Ampedus pomonae* (Stephens, 1830)									1	1.4	1
*Ampedus pomorum* (Herbst, 1784)	8	6.7							3	2.8	11
*Ampedus praeustus* (Fabricius, 1792)											
*Ampedus sanguinolentus* (Schrank, 1776)	5	5.6			2	4.0			3	2.8	10
*Cidnopus aeruginosus* (G.-A. Olivier, 1790)			1	5.0							1
*Ctenicera pectinicornis* (Linnaeus, 1758)									1	1.4	1
*Dalopius marginatus* (Linnaeus, 1758)							1	1.8			1
*Danosoma fasciatum* (Linnaeus, 1758)			1	5.0							1
*Dicronychus equiseti* (Herbst, 1784)	8	5.6	1	5.0							9
*Hemicrepidius niger* (Linnaeus, 1758)					2	4.0					2
*Limonius minutus* (Linnaeus, 1758)									3	4.3	3
*Melanotus castanipes* (Paykull, 1800)			1	5.0					1	1.4	2
*Melanotus villosus* (Geoffroy, 1785)	1	1.1					3	5.4	3	4.3	7
*Mosotalesus impressus* (Fabricius, 1792)	1	1.1									1
*Pristilophus cruciatus* (Linnaeus, 1758)			1	5.0							1
*Prosternon tessellatum* (Linnaeus, 1758)	27	22.2			25	16.0	44	42.9	98	37.1	194
*Selatosomus aeneus* (Linnaeus, 1758)	1	1.1									1
**Histeridae**											
*Gnathoncus buyssoni* (Auzat, 1917)									1	1.4	1
Margarinotus striola (C.R. Sahlberg, 1819)									1	1.4	1
*Paromalus parallelepipedus* (Herbst, 1791)									2	1.4	2
*Saprinus rugifer* (Paykull, 1809)					1	2.0					1
**Hydrochidae**											
*Hydrochus brevis* (Herbst, 1793)			1	5.0							1
**Staphylinidae**											
*Oiceoptoma thoracicum* (Linnaeus, 1758)			6	15.0	11	14.0			28	17.1	45
Staphylinidae sp.	13	10	38	45.0	36	40.0	18	23.2	195	34.3	300
*Quedius dilatatus* (Fabricius, 1787)							4	7.1			4
**Scarabaeidae**											
*Amphimallon altaicum* (Mannerheim, 1825)					1	2.0					1
*Cetonia aurata* (Linnaeus, 1758)	155	48.9	1	5.0	36	26.0	490	57.1	55	40.0	737
*Gnorimus variabilis* (Linnaeus, 1758)											
*Oxythyrea funesta* (Poda von Neuhaus, 1761)	8	8.9			1	2.0	139	66.1	6	7.1	154
*Phyllopertha horticola* (Linnaeus, 1758)	3	3.3					4	7.1	16	10.0	23
*Protaetia fieberi* (Kraatz, 1880)	3	2.2			5	8.0	13	14.3	22	10.0	43
*Protaetia marmorata* (Fabricus, 1792)	12	12.2	2	10.0	6	10.0	47	33.9	95	47.1	162
*Protaetia speciosissima* (Scopoli, 1786)							1	1.8			1
*Protaetia cuprea volhyniensis* (Gory & Percheron, 1833)	139	56.7			9	18.0	303	71.4	91	40.0	542
*Serica brunnea* (Linnaeus, 1758)					1	2.0					1
*Trichius fasciatus* (Linnaeus, 1758)	1	1.1					6	10.7	9	8.6	16
**Dermestidae**											
*Attagenus schaefferi* (Herbst, 1792)	2	1.1							4	5.7	6
*Dermestes lardarius* (Linnaeus, 1758)	1	1.1									1
*Dermestes murinus* (Linnaeus, 1758)	1	1.1					1	1.8			2
*Trogoderma glabrum* (Herbst, 1783)	3	3.3			1	2.0			4	4.3	8
**Cleridae**											
*Thanasimus formicarius* (Linnaeus, 1758)									9	8.6	9
*Trichodes apiarius* (Linnaeus, 1758)	3	3.3			2	4.0	121	55.4	13	15.7	139
**Melyridae**											
*Charopus flavipes* (Paykull, 1798)									1	1.4	1
*Cordylepherus viridis* (Fabricius, 1787)	1	1.1	1	5.0	1	2.0			6	7.1	9
*Dasytes fusculus* (Illiger, 1801)					1	2.0					1
*Dasytes niger* (Linnaeus, 1761)	2	2.2	2	5.0	22	20.0	1	1.8	14	15.7	41
*Dolichosoma lineare* (P. Rossi, 1794)					5	2.0			1	1.4	6
*Malachius aeneus* (Linnaeus, 1758)									1	1.4	1
*Malachius bipustulatus* (Linnaeus, 1758)	1	1.1	3	15.0	6	6.0			3	4.3	13
**Mordellidae**											
*Mordella* sp.	1	1.1					3	1.8	6	5.7	10
*Mordellistena humeralis* (Linnaeus, 1758)			2	10.0							2
*Mordellistena* sp.					1	2.0			1	1.4	2
*Tomoxia bucephala* (A. Costa, 1854)	1	1.1							3	4.3	4
*Variimorda* sp.					1	2.0					1
**Scraptiidae**											
*Anaspis frontalis* (Linnaeus, 1758)					17	10.0			2	2.8	19
*Anaspis thoracica* (Linnaeus, 1758)	1	1.1									1
**Oedemeridae**											
*Chrysanthia geniculata* (W.L.E. Schmidt, 1846)							9	8.9	6	2.8	15
*Chrysanthia viridissima* (Linnaeus, 1758)	92	8.9			1	2.0	214	60.7	7	7.1	314
*Oedemera femorata* (Scopoli, 1763)					2	4.0	1	1.8	5	7.1	8
*Oedemera virescens* (Linnaeus, 1767)	1	1.1			2	4.0			4	5.7	7
**Anthicidae**											
*Notoxus monoceros* (Linnaeus, 1761)	2	2.2	4	15.0	1	2.0	3	1.8	41	8.6	51
**Melandryidae**											
*Dircaea quadriguttata* (Paykull, 1798)			1	5.0							1
**Mycetophagidae**											
*Litargus connexus* (Geoffroy, 1785)									2	1.4	2
**Tenebrionidae**											
*Bolitophagus reticulatus* (Linnaeus, 1767)					1	2.0			1	1.4	2
*Isomira murina* (Linnaeus, 1758)					2	2.0	15	16.1			17
*Lagria hirta* (Linnaeus, 1758)	1	1.1			1	2.0	1	1.8	4	5.7	7
*Pseudocistela ceramboides* (Linnaeus, 1758)	1	1.1									1
**Latridiidae**											
*Corticaria* sp.			1	5.0							1
**Coccinellidae**											
*Ceratomegilla notata* (Laicharting, 1781)									3	4.3	3
*Coccinella hieroglyphica* (Linnaeus, 1758)					1	2.0					1
*Coccinella magnifica* (L. Redtenbacher, 1843)							11	16.1	2	2.8	13
*Coccinella quinquepunctata* (Linnaeus, 1758)			1	5.0							1
*Coccinella septempunctata* (Linnaeus, 1758)	16	15.6			10	12.0	17	21.4	12	14.3	55
*Coccinula quatuordecimpustulata* (Linnaeus, 1758)									4	4.3	4
*Exochomus quadripustulatus* (Linnaeus, 1758)					1	2.0					1
*Hippodamia variegata* (Goeze, 1777)			1	5.0			1	1.8			2
*Hyperaspis concolor* (Suffrian, 1843)					2	4.0					2
*Platynaspis luteorubra* (Goeze, 1777)					2	4.0					2
*Propylea quatuordecimpunctata* (Linnaeus, 1758)					1	2.0			1	1.4	2
*Psyllobora vigintiduopunctata* (Linnaeus, 1758)	2	2.2			2	4.0	1	1.8	3	4.3	8
*Subcoccinella vigintiquatuorpunctata* (Linnaeus, 1758)	1	1.1									1
*Tytthaspis gebleri* (Mulsant, 1850)									1	1.4	1
**Erotylidae**											
*Triplax russica* (Linnaeus, 1758)	2	2.2					1	1.8	1	1.4	4
*Triplax rufipes* (Fabricius, 1787)									1	1.4	1
**Monotomidae**											
*Rhizophagus fenestralis* (Linnaeus, 1758)			1	5.0					13	5.7	14
**Nitidulidae**											
*Carpophilus hemipterus* (Linnaeus, 1758)	2	2.2			2	4.0					4
*Carpophilus marginellus* (Motschulsky, 1858)					2	2.0			1	1.4	3
*Carpophilus* sp.									2	2.8	2
*Cryptarcha strigata* (Fabricius, 1787)	53	21.1	10	45.0	23	30.0	50	35.7	364	40.0	500
*Cryptarcha undata* (G.-A. Olivier, 1790)					4	6.0	2	3.6			6
*Cychramus luteus* (Fabricius, 1787)					1	2.0			3	2.8	4
*Epuraea guttata* (G.-A. Olivier, 1811)					1	2.0			9	4.3	10
*Epuraea* sp.	54	20	37	45.0	32	28.0	5	5.4	108	21.4	236
*Glischrochilus grandis* (Tournier, 1872)	109	33.3	38	45.0	53	28.0	142	26.8	1059	45.7	1401
*Glischrochilus hortensis* (Geoffroy, 1785)	25	10	123	55.0	110	26.0	2	3.6	39	27.1	299
*Glischrochilus quadriguttatus* (Fabricius, 1777)	2	2.2	5	20.0	1	2.0					8
*Glischrochilus quadripunctatus* (Linnaeus, 1758)	5	5.6	4	20.0					11	12.9	20
*Glischrochilus quadrisignatus* (Say, 1835)	1	1.1	1	5.0	10	10.0	1	1.8	3	2.8	16
*Ipidia binotata* (Reitter, 1875)			1	5.0	1	2.0					2
*Meligethes* sp.			1	5.0					1	1.4	2
*Omosita depressa* (Linnaeus, 1758)									1	1.4	1
*Omosita japonica* (Reitter, 1874)					1	2.0					1
*Soronia grisea* (Linnaeus, 1758)	107	27.8	135	75.0	105	36.0	10	12.5	96	30.0	453
*Soronia punctatissima* (Illiger, 1794)							1	1.8			1
**Cucujidae**											
*Pediacus depressus* (Herbst, 1797)									1	1.4	1
**Silvanidae**											
*Ahasverus advena* (Waltl, 1834)									1	1.4	1
*Psammoecus bipunctatus* (Fabricius, 1792)					1	2.0					1
*Silvanus bidentatus* (Fabricius, 1792)									1	1.4	1
**Phalacridae**											
*Phalacrus* sp.					1	2.0					1
**Laemophloeidae**											
*Cryptolestes pusillus* (Schoenherr, 1817)									3	4.3	3
**Cerambycidae**											
*Aegomorphus clavipes* (Schrank, 1781)											
*Acanthocinus aedilis* (Linnaeus, 1758)									3	4.3	3
*Agapanthia villosoviridescens* (De Geer, 1775)					1	2.0					1
*Anastrangalia reyi* (L. Heyden, 1889)							10	10.7	1	1.4	11
*Arhopalus rusticus* (Linnaeus, 1758)							1	1.8			1
*Aromia moschata* (Linnaeus, 1758)	4	4.4	18	20.0	19	22.0	12	12.5	5	5.7	58
*Asemum striatum* (Linnaeus, 1758)							4	5.4			4
*Chlorophorus herbstii* (Brahm, 1790)							1	1.8			1
*Dinoptera collaris* (Linnaeus, 1758)							1	1.8			1
*Leptura quadrifasciata* (Linnaeus, 1758)	60	14.4	4	20.0	3	6.0	118	55.4	121	41.4	306
*Leptura thoracica* (Creutzer, 1799)	10	11.1	1	5.0			1	1.8	17	11.4	29
*Lepturalia nigripes* (De Geer, 1775)	26	15.6					36	25	32	24.3	94
*Lepturobosca virens* (Linnaeus, 1758)							1	1.8			1
*Monochamus galloprovincialis pistor* (Germar, 1818)									1	1.4	1
*Necydalis major* (Linnaeus, 1758)					1	2.0	1	1.8			2
*Nivellia sanguinosa* (Gyllenhal, 1827)									1	1.4	1
*Pachyta quadrimaculata* (Linnaeus, 1758)	2	2.2					4	5.4	10	5.7	16
*Plagionotus arcuatus* (Linnaeus, 1758)									1	1.4	1
*Pseudovadonia livida* (Fabricius, 1776)									1	1.4	1
*Purpuricenus globulicollis* (Dejean, 1839)									1	1.4	1
*Purpuricenus kaehleri* (Linnaeus, 1758)	1	1.1			1	2.0	3	5.4			5
*Rhagium inquisitor* (Linnaeus, 1758)									2	2.8	2
*Rhagium mordax* (De Geer, 1775)	35	14.4	2	10.0	2	4.0	3	5.4	64	22.9	106
*Rutpela maculata* (Poda von Neuhaus, 1761)	2	2.2					46	37.5	1	1.4	49
*Stenocorus meridianus* (Linnaeus, 1758)									1	1.4	1
*Stenurella bifasciata* (O.F. Müller, 1776)							17	12.5			17
*Stenurella melanura* (Linnaeus, 1758)	1	1.1					42	25	1	1.4	44
*Stictoleptura maculicornis* (De Geer, 1775)							2	3.6	2	2.8	4
*Stictoleptura rubra* (Linnaeus, 1758)							6	10.7	3	2.8	9
*Stictoleptura variicornis* (Dalman, 1817)							1	1.8			1
*Strangalia attenuata* (Linnaeus, 1758)					1	2.0	20	32.1	1	1.4	22
*Xylotrechus antilope* (Schoenherr, 1817)	1	1.1									1
*Xylotrechus arvicola* (Olivier, 1795)							1	1.8			1
*Xylotrechus capricornus* (Gebler, 1830)	1	1.1							1	1.4	2
*Xylotrechus rusticus* (Linnaeus, 1758)									4	5.7	4
**Chrysomelidae**											
*Altica* sp.			1	5.0					1	1.4	2
*Bromius obscurus* (Linnaeus, 1758)							1	1.8	1	1.4	2
*Cassida vittata* (Villers, 1789)			1	5.0							1
*Crepidodera fulvicornis* (Fabricius, 1792)			1	5.0							1
*Cryptocephalus flavipes* (Fabricius, 1781)							1	1.8			1
*Cryptocephalus sericeus* (Linnaeus, 1758)							1	1.8			1
*Galerucella lineola* (Fabricius, 1781)					1	2.0					1
*Gonioctena linnaeana* (Schrank, 1781)			5	15.0							5
*Gonioctena viminalis* (Linnaeus, 1758)									1	1.4	1
*Hypocassida subferruginea* (Schrank, 1776)	1	1.1							1	1.4	2
*Lema cyanella* (Linnaeus, 1758)					1	2.0					1
*Lilioceris merdigera* (Linnaeus, 1758)									1	1.4	1
*Lochmaea caprea* (Linnaeus, 1758)			1	5.0	2	4.0					3
*Spermophagus sericeus* (Geoffroy, 1785)									1	1.4	1
**Attelabidae**											
*Deporaus betulae* (Linnaeus, 1758)									1	1.4	1
**Anthribidae**											
*Platystomos albinus* (Linnaeus, 1758)									1	1.4	1
*Tropideres albirostris* (Schaller, 1783)					4	6.0			2	2.8	6
**Brentidae**											
*Eutrichapion ervi* (Kirby, 1808)									1	1.4	1
*Protapion interjectum* (Desbrochers des Loges, 1895)	1	1.1									1
*Protapion fulvipes* (Geoffroy, 1785)									1	1.4	1
**Curculionidae**											
*Anisandrus dispar* (Fabricius, 1792)	25	7.8	282	45.0	100	26.0	8	5.4	208	21.4	623
*Anthonomus phyllocola* (Herbst, 1795)	1	1.1							1	1.4	2
*Anthonomus pomorum* (Linnaeus, 1758)			1	5.0							1
*Anthonomus rubi* (Herbst, 1795)					1	2.0					1
*Cleopomiarus distinctus* (Boheman, 1845)					1	2.0					1
*Dorytomus dorsalis* (Linnaeus, 1758)	1	1.1									1
*Hylurgus ligniperda* (Fabricius, 1787)	1	1.1									1
*Hypera conmaculata* (Herbst, 1795)					1	2.0					1
*Larinus obtusus* (Gyllenhal, 1835)							1	1.8			1
*Larinus sturnus* (Schaller, 1783)									1	1.4	1
*Larinus turbinatus* (Gyllenhal, 1835)									1	1.4	1
*Miarus ajugae* (Herbst, 1795)	1	1.1							2	2.8	3
*Mononychus punctumalbum* (Herbst, 1784)					1	2.0					1
*Otiorhynchus ovatus* (Linnaeus, 1758)	1	1.1									1
*Phyllobius argentatus* (Linnaeus, 1758)									2	2.8	2
*Phyllobius maculicornis* (Germar, 1823)	4	2.2							1	1.4	5
*Phyllobius oblongus* (Linnaeus, 1758)			1	5.0							1
*Phyllobius pomaceus* (Gyllenhal, 1834)					3	6.0					3
*Phyllobius pyri* (Linnaeus, 1758)	32	15.6	1	5.0	9	10.0			4	5.7	46
*Pissodes castaneus* (De Geer, 1775)	11	6.7									11
*Pissodes pini* (Linnaeus, 1758)									1	1.4	1
*Polydrusus tereticollis* (De Geer, 1775)	1	1.1									1
*Sitona lineatus* (Linnaeus, 1758)			1	5.0							1
*Strophosoma capitatum* (De Geer, 1775)	6	6.7									6
*Tachyerges decoratus* (Germar, 1821)			1	5.0					1	1.4	2
*Xyleborinus saxesenii* (Ratzeburg, 1837)					1	2.0					1
**Total**	**1275**		**751**		**747**		**2040**		**3093**		**7906**
